# 34例原发性甲状腺淋巴瘤的临床特征及预后分析

**DOI:** 10.3760/cma.j.cn121090-20240220-00068

**Published:** 2024-05

**Authors:** 磊 杨, 立洁 曾, 进 叶, 立强 魏, 佳 丛, 鑫 李, 娜 姚, 晶 杨, 赫男 王, 黎玮 吕, 轶苹 吴, 亮 王

**Affiliations:** 首都医科大学附属北京同仁医院血液科，北京 100730 Department of Hematology, Beijing Tongren Hospital Affiliated to Capital Medical University, Beijing 100730, China

**Keywords:** 原发性甲状腺淋巴瘤, 病理诊断, 疗效评估, 危险因素, Primary thyroid lymphoma, Pathological diagnosis, Treatment efficacy evaluation, Risk factors

## Abstract

**目的:**

探讨原发性甲状腺淋巴瘤（Primary thyroid lymphoma，PTL）的临床特征、诊断、治疗及预后。

**方法:**

回顾性分析2010年9月至2023年2月北京同仁医院收治的34例新诊断PTL患者的临床和病理资料，采用Kaplan-Meier生存曲线及Log-rank检验进行生存分析，预后影响因素分析采用单因素Cox回归模型。

**结果:**

34例PTL患者均以颈部肿物为首发临床表现，男9例，女25例，病理诊断为弥漫大B细胞淋巴瘤（DLBCL）、黏膜相关淋巴组织（MALT）淋巴瘤的患者分别为29例、5例。DLBCL患者中，6例伴有B症状，17例美国东部肿瘤协作组（ECOG）评分≥2分，Ann Arbor分期为Ⅰ～Ⅱ期21例，Ⅲ～Ⅳ期8例，肿块长径≥10 cm 4例，14例合并桥本甲状腺炎；27例接受化疗，完全缓解（CR）21例，部分缓解（PR）2例，疾病进展6例；5年无进展生存率、总生存率分别为78.9％、77.4％；单因素生存分析显示，B症状、肿块长径≥10 cm、Ann Arbor Ⅲ～Ⅳ期是影响患者预后的重要因素（*P*<0.05）。MALT淋巴瘤患者均为Ⅰ～Ⅱ期、ECOG评分0～1分、无B症状，患者均进行了手术切除，CR 4例，PR 1例。

**结论:**

PTL常见于合并桥本甲状腺炎的女性患者，病理类型多为B细胞淋巴瘤，治疗上以化疗为主，放疗及手术为辅，预后相对较好。

## Abstract (en)

**Objective:**

To investigate the clinical characteristics, diagnosis, treatment, and prognosis of primary thyroid lymphoma（PTL）.

**Methods:**

A retrospective analysis was conducted on the clinical and pathological data of 34 newly diagnosed PTL patients admitted to Beijing Tongren Hospital from September 2010 to February 2023. The Kaplan-Meier survival curve and Log-rank test were used for survival analysis, and the Cox regression model was applied for univariate analysis of prognostic factors.

**Results:**

All 34 PTL patients presented with cervical mass as the initial clinical manifestation. There were 9 males and 25 females. The pathological diagnosis was diffuse large B-cell lymphoma（DLBCL）in 29 patients and mucosa-associated lymphoid tissue（MALT）lymphoma in 5 patients. Among the DLBCL patients, 6 had B symptoms, 17 had an Eastern Cooperative Oncology Group（ECOG）score of ≥2, the Ann Arbor staging was stage Ⅰ–Ⅱ in 21 cases and stage Ⅲ–Ⅳ in 8 cases, the tumor diameter was ≥10 cm in 4 cases, and 14 had concurrent Hashimoto thyroiditis; 27 cases received chemotherapy, with 21 cases achieving complete remission（CR）, 2 cases partial remission（PR）, and 6 cases of disease progression; the 5-year progression-free survival and overall survival rates were 78.9% and 77.4%, respectively; univariate survival analysis showed that B symptoms, tumor diameter ≥10 cm, and Ann Arbor stage Ⅲ–Ⅳ were significant factors affecting patient prognosis（*P*<0.05）. MALT lymphoma patients were all in stages Ⅰ–Ⅱ, had an ECOG score of 0-1, and were without B symptoms. All patients underwent surgical resection, with 4 cases achieving CR and 1 case PR.

**Conclusion:**

PTL is more common in females with concurrent Hashimoto thyroiditis, with the majority of pathological types being B-cell lymphoma. The main treatment is chemotherapy, supplemented by radiotherapy and surgery, and the prognosis is relatively favorable.

原发性甲状腺淋巴瘤（Primary thyroid lymphoma，PTL）是指发生在甲状腺的淋巴瘤，可伴有局部淋巴结或临近组织器官的浸润，常以颈部肿物、吞咽困难等为首发临床表现。PTL发病率低，占甲状腺恶性肿瘤的1％～5％，占结外非霍奇金淋巴瘤（NHL）的2.5％～7％[1]。PTL可表现为多种病理亚型，但临床上仍以弥漫大B细胞淋巴瘤（DLBCL）最为常见，占50％～70％[2]。该病缺乏特异性，临床表现容易与桥本甲状腺炎（Hashimoto thyroiditis，HT）及甲状腺癌相混淆。PTL患者对化疗及放疗比较敏感，随着治疗方法的发展，患者预后得到明显改善。本研究总结我中心诊治的34例PTL患者的临床资料，旨在探究PTL的临床特点、病理特征、治疗及预后，以期提高对PTL的认识和诊治水平。

## 病例与方法

1. 病例资料：回顾性分析2010年9月到2023年2月期间北京同仁医院收治的34例新诊断未经治疗的PTL患者的临床资料。原发甲状腺淋巴瘤（PTL）的诊断标准：①年龄大于14岁；②病理类型参照2016年的WHO造血和淋巴系统肿瘤分类标准[3]进行分类，由我院病理科医师经组织学和免疫组化检查确诊为NHL；③肿瘤起源于甲状腺，并排除由于转移或直接延伸而侵犯甲状腺的肿瘤。病理诊断DLBCL 29例，黏膜相关淋巴组织（MALT）淋巴瘤5例。临床分期仍采用Ann Arbor分期，预后评估参照国际预后指数（IPI）评分标准[4]。双表达淋巴瘤定义为同时伴有c-Myc（≥40％）和Bcl-2（≥50％）蛋白过表达的DLBCL，三表达淋巴瘤定义为在双表达淋巴瘤的基础上，合并Bcl-6（≥50％）蛋白过表达的DLBCL[3]。

2. 治疗方案：29例DLBCL患者中，10例行甲状腺切除术，27例进行化疗。一线化疗采用R-CHOP（利妥昔单抗+环磷酰胺+长春新碱+表柔比星+醋酸泼尼松）方案或R-EPOCH（利妥昔单抗+环磷酰胺+长春新碱+表柔比星+依托泊苷+醋酸泼尼松）方案；二线化疗采用R-EPOCH方案或ZR-CHOP（泽布替尼+利妥昔单抗+环磷酰胺+长春新碱+表柔比星+醋酸泼尼松）方案。5例MALT淋巴瘤患者行手术切除。

3. 疗效评价及随访：采用甲状腺彩色多普勒超声，颈部增强磁共振成像或CT并结合PET-CT评估局部及全身病灶缓解情况。在治疗结束后6～8周进行疗效评估，评估采用2007年Cheson淋巴瘤疗效评价修订标准，分为完全缓解（CR）、部分缓解（PR）、疾病稳定（SD）和疾病进展（PD）。随访截止时间为2024年1月，中位随访时间为46个月，采用门诊复查、住院治疗及电话随访。患者的总生存（OS）期定义为从确诊之日起到发生任何原因死亡或随访终点的时间，无进展生存（PFS）期定义为确诊之日起至出现进展、死亡或随访终止的时间。

4. 统计学处理：计数资料采用例数（构成比）描述。本研究采用R4.0.3软件进行统计学分析。生存分析采用Kaplan-Meier生存曲线，组间比较采用Log-rank检验。预后影响因素分析采用单因素Cox回归模型。*P*<0.05为差异有统计学意义。

## 结果

1. 基本临床特征：PTL患者共34例，男9例，女25例，中位年龄63（36～86）岁。其中，单纯累及甲状腺26例，伴颈部淋巴结、食管及气管等周围组织累及8例。免疫组化显示c-Myc蛋白表达阳性患者6例。病理诊断为DLBCL的患者29例，男6例、女23例，中位年龄64（36～86）岁，就诊时均以颈前肿物为首发临床表现，CT表现见图1。29例患者中伴有声音嘶哑者6例（20.7％），检查发现为压迫喉返神经，声带麻痹所致；肿块压迫导致呼吸困难及吞咽困难8例（27.6％）；6例（20.7％）患者疾病初期伴有B症状（盗汗、发热及体重减轻）；17例（58.6％）美国东部肿瘤协作组（ECOG）评分≥2分；Ann Arbor Ⅰ～Ⅱ期21例（72.4％），Ⅲ～Ⅳ期8例（27.6％）；实验室检查提示LDH升高13例（44.8％），β_2_微球蛋白（β_2_-MG）升高5例（17.2％）；IPI评分0～1分10例（34.5％）、2～5分19例（65.5％）；甲状腺功能检测及超声多普勒结果显示合并HT 14例（48.3％）；甲状腺彩色多普勒超声检查提示病灶以低回声结节为主要表现者18例（62.1％）、以弥漫性低回声病变为主要表现者8例（27.6％）；结合手术及影像资料评估肿块长径≥10 cm患者4例（13.8％）。5例MALT淋巴瘤患者中，男3例，女2例，中位年龄62（57～76）岁，均为Ann Arbor分期为Ⅰ～Ⅱ期，ECOG评分为0～1分，无B症状及明显呼吸道压迫，LDH及β_2_-MG正常。

**图1:**
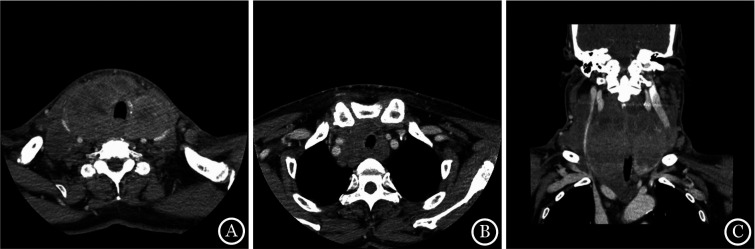
原发性甲状腺淋巴瘤患者头颈部CT检查 **注** A：甲状腺明显增大，边界不清，密度不均，气道受压变窄；B：病变累及上纵隔，与食管及纵隔内肿大淋巴结分界不清；C：病变包绕双侧颈总动脉及颈内静脉

2. 病理特征：所有患者行病理及免疫组织化学检查，确诊DLBCL 29例（85.3％），其中经超声引导下穿刺活检证实19例（65.5％），手术切除活检10例（34.5％）。采用Hans免疫分型法进一步将29例DLBCL患者分为生发中心B细胞（GCB）和非GCB（non-GCB）亚型，其中GCB有8例（27.6％），non-GCB有21例（72.4％）。免疫组化结果显示Ki-67≥80％ 15例（51.7％），在完成Bcl-2、Bcl-6及c-Myc免疫组化检测的15例患者中，确定双/三表达4例（26.6％）。MALT淋巴瘤患者5例（17.2％）全部为甲状腺手术切除病理证实，其中2例先予以超声引导下穿刺活检，因组织较少形态不典型无法明确，后行手术切除病理证实。

3. 治疗方案及疗效评估：29例DLBCL患者中，进行手术切除的共10例（34.5％）。除1例患者放弃治疗，1例患者单纯放疗外，27例均接受化疗（单纯化疗16例，手术+化疗10例，手术+化疗+放疗1例）。接受一线R-CHOP方案的患者共25例，R-EPOCH方案2例，二线予R-EPOCH方案2例，ZR-CHOP方案2例（均为双/三表达）。1例患者三线接受嵌合抗原受体T（CAR-T）细胞治疗。化疗患者在至少完成4个疗程的治疗后评估疗效：获得CR患者21例（72.4％），PR患者2例（6.9％），PD患者6例（20.7％）。5例MALT患者全部行手术切除，其中术后联合放疗1例，联合化疗1例（R-CHOP方案），评估疗效为CR患者4例（80％），PR患者1例（20％）。

4. 远期生存情况及预后分析：死亡6例，全部为DLBCL患者，除1例临床分期为Ⅱ期外，其余5例均由于病变为局部广泛累及（甲状腺、颈部淋巴结、气管、食管、咽喉部）而诊断为Ⅳ期，其中3例在治疗前已行气管切开术缓解症状；1例患者放弃治疗死亡，另5例患者一线接受R-CHOP或R-EPOCH方案化疗，但分别于3～5个疗程后出现PD导致死亡。5例MALT淋巴瘤患者全部存活。

DLBCL患者中位随访46个月，5年PFS率、OS率分别为78.9％、77.4％。其中Ann Arbor Ⅰ～Ⅱ期患者5年的PFS率、OS率分别为95.2％、94.4％，Ⅲ～Ⅳ期患者5年的PFS率和OS率均为37.5％，中位PFS期、OS期分别为7个月、12个月，明显低于Ⅰ～Ⅱ期患者（*P*值均<0.001）；肿块长径<10 cm患者5年的PFS率、OS率分别为88.0％、87.3％，而肿块长径≥10 cm患者中位PFS期仅为6个月，OS期为12个月；IPI评分为0～1分患者5年的PFS率和OS率均为100％，2～5分患者5年的PFS率、OS率分别为67.4％、65.2％（*P*值均<0.001）。对29例DLBCL患者进行单因素预后分析，影响患者PFS的不良预后因素包括：B症状、肿块长径≥10 cm、Ann Arbor Ⅲ～Ⅳ期、ECOG评分≥2分；影响患者的OS的不良预后因素包括B症状、肿块长径≥10 cm、Ann Arbor Ⅲ～Ⅳ期（表1）。

**表1:** 病理诊断为弥漫性大B细胞淋巴瘤的原发性甲状腺淋巴瘤患者的单因素预后分析

危险因素	无进展生存		总生存	
	*HR*（95%*CI*）	*P*值	*HR*（95%*CI*）	*P*值
年龄>60岁	0.498（0.100～2.473）	0.394	0.498（0.100～2.471）	0.394
性别	0（0～Inf）	0.999	0（0～Inf）	0.999
ECOG评分≥2分	144 646 915.875（25 861 895.005～809 017 679.018）	<0.001	654 315 977.520（0～Inf）	0.999
Ann Arbor Ⅲ～Ⅳ期	16.966（1.971～146.069）	0.010	16.736（1.943～144.158）	0.010
LDH水平升高	2.963（0.541～16.243）	0.211	2.840（0.520～15.510）	0.228
肿块长径≥10 cm	10.766（2.098～55.240）	0.004	14.782（2.824～77.376）	0.001
B症状	5.015（1.005～25.013）	0.049	5.574（1.119～27.761）	0.036
IPI评分2～5分	408 589 769.320（0～Inf）	0.998	408 353 125.540（0～Inf）	0.998
GCB来源	2.135（0.249～18.292）	0.489	1.792（0.209～15.364）	0.595
Ki-67≥80％	0.973（0.915～1.035）	0.388	0.978（0.920～1.041）	0.489
双/三表达淋巴瘤	2.207（0.257～18.944）	0.470	1.959（0.229～16.786）	0.540
手术治疗	0.208（0.038～1.142）	0.071	0.245（0.045～1.341）	0.105

**注** ECOG：美国东部肿瘤协作组；IPI：国际预后指数；GCB：生发中心B细胞；Inf：趋于无限大

## 讨论

PTL在女性患者中的发病率是男性的2～8倍[2],[5]。本研究中女性患者例数为男性的3.8倍。一项Meta分析纳入了38项临床研究，共报告了1 346例PTL患者，其中合并HT（甲状腺的自身抗体阳性，结合病史查体或组织学证据证实）患者占78.9％[6]。HT患者在长期慢性炎症的刺激下，PTL的发生相对风险增加60～80倍[7]。本研究DLBCL合并HT患者占48.3％，提示两者间存在着相关性。

PTL的诊断主要通过手术切除或彩色多普勒引导下细针或粗针穿刺获取病理。Meta分析显示，细针穿刺细胞学检查敏感度仅为48％。相比之下，粗针穿刺活检技术准确率高，尤其对于压迫症状重、无法耐受手术的患者更加方便安全[8]。本研究中，DLBCL患者中19例行粗针穿刺活检并均得取阳性病例结果，但MALT淋巴瘤5例患者当中2例无法确诊，可能与惰性淋巴瘤肿瘤细胞体积偏小、辨识度低有关。

在PTL病理亚型中，B细胞型NHL占绝大多数。DLBCL最常见，占50％～80％，其次为MALT淋巴瘤约占20％。其他类型较为罕见[9]–[12]。本研究中DLBCL占85.3％，为最常见亚型。Hans模型是目前DLBCL常用的病理分型[13]，本研究29例患者中，non-GCB亚型更为常见，占72.4％，与Yi等[14]的报道相似。30％～35％的DLBCL患者表达c-Myc，20％～35％同时表达Bcl-2，为双表达淋巴瘤，患者预后不良[15]。本研究中15例患者完成免疫组化Bcl-2、Bcl-6及c-Myc检测，确定双/三表达淋巴瘤4例（26.6％），2例患者死于PD，但因例数较少，在单因素分析中未显示其是影响PFS及OS的预后因素。分子生物学方面，既往研究中对甲状腺DLBCL的靶向测序结果显示，TET2、KMT2D、TP53、GNA13、KMT2C是其中常见的高突变基因，并且伴有TP53突变的患者预后不佳[16]。另外，有学者发现，Wnt5a蛋白及其受体Ror2与PTL的发生有关[17]。多项国内外研究显示IPI评分系统对于DLBCL预后判断有重要价值，而这同样适用于PTL患者[14],[18]–[19]。本研究中IPI评分2～5分患者5年PFS及OS率低于0～1分患者，但单因素分析未发现IPI评分对生存有影响，可能与样本量较少有关。此外，本研究通过单因素生存分析发现影响PFS及OS的因素包括Ann Arbor分期、B症状及肿块长径≥10 cm。在Zhu等[5]对2 215例PTL患者的预后分析模型中，报道了年龄大于60岁及Ann Arbor分期为影响患者OS的因素，但该研究并未将B症状及肿块长径纳入分析模型中。

治疗上，应根据患者的病理类型、分期、危险分层及对治疗的反应等因素综合选择。在病理类型为DLBCL患者中，仍以包含利妥昔单抗的化疗方案为主，部分联合放疗及局部手术治疗。Yi等[14]在对于病理证实DLBCL的58例PTL患者的多中心、回顾性研究中发现，利妥昔单抗治疗的患者5年OS率与PFS率明显高于未使用的患者。在本研究中，DLBCL患者均采用了包含利妥昔单抗的治疗方案，患者的5年OS率与PFS率和文献中报道相近，肯定了CD20单抗在该类患者中的治疗价值。对于中高危患者，尤其是双/三表达淋巴瘤、一线治疗后疾病进展或复发患者，推荐采用更强的二线方案、临床试验。目前沿用了NCCN指南中对于DLBCL推荐的治疗方案，新型疗法如CAR-T细胞治疗及双特异性抗体等可以提高此类患者缓解率，二线缓解后可行造血干细胞移植巩固，但由于该病发病率低，缺乏大规模前瞻性临床研究，尚存争议，需更高级别的证据支持。本研究中1例PD患者进行CAR-T细胞治疗后再次获得CR。对于MALT淋巴瘤患者，如病变局限于甲状腺，则推荐使用手术治疗、局部放疗或免疫治疗等方法[20]–[22]。Saito等[23]在对病理结果为MALT淋巴瘤、分期为IE期的PTL患者的回顾性研究中发现，对于这些患者，手术切除与局部放疗的疗效相近，同时手术切除可以避免因放疗带来的疼痛、长期口干等不良反应。本研究中病理诊断为MALT淋巴瘤的患者，均接受了手术治疗且预后良好，但因纳入病例较少，其疗效仍有待观察。另外，由于PTL可能导致急性呼吸道压迫症状，此时进行手术解除气道梗阻是必要的。
